# Rare Cause of Stricture Esophagus—Sarcoma: A Case Report and Review of the literature

**DOI:** 10.1155/2011/192423

**Published:** 2011-10-13

**Authors:** S. Patricia, Das Saikat, B. Rajesh, I. Rajesh, B. Selvamani, John Subhashini

**Affiliations:** Department of Radiation Oncology, Dr. Ida Scudder Cancer Center, Christian Medical College, Vellore 632004, India

## Abstract

Adenocarcinoma and squamous cell carcinoma account for the vast majority of oesophageal malignancies. Other malignancies known to occur in the oesophagus include melanoma, sarcoma, and lymphoma. Among the sarcomas, carcinosarcoma is the commonest with both carcinomatous and sarcomatous elements followed by leiomyosarcoma of mesenchymal origin. Other sarcomas reported in the literature are liposarcoma, synovial sarcoma, myxofibrosarcoma, Ewing's sarcoma, granulocytic sarcoma, histiocytic sarcoma, schwannoma rhabdomyosarcoma, and epithelioid sarcoma. We report a case of malignant spindle cell tumour of oesophagus. Sarcomas of esophagus present as a polypoid exophytic soft tissue mass. Our patient presented with a stricture which is a rare presentation. Locally aggressive treatment with surgery is beneficial, and local palliative treatment including radiotherapy is worthwhile.

## 1. Introduction

Ninety-five percent of esophageal malignancies are of epithelial origin: adenocarcinoma and squamous cell carcinoma [1]. Sarcomas are a rare entity along with melanoma and lymphoma. Varied histologies of sarcomas are reported such as carcinosarcoma (both epithelial and mesenchymal elements), leiomyosarcoma [2], synovial sarcoma [3], myxofibrosarcoma [4], Ewing's sarcoma [5], granulocytic sarcoma [6], histiocytic sarcoma [7], schwannoma [8], rhabdomyosarcoma [9], and epithelioid sarcoma. Sarcomas present as an exophytic polypoidal mass. We present a case of stricture esophagus turned out to be malignant spindle cell tumor. 

## 2. Case Report

Sixty-three-year-old man who was a dentist by profession from West Bengal presented in March 2005 with complaints of dysphagia to solids for 12 yrs aggravated for 1 month. He had no loss of appetite but had loss of weight. His physical examination was unremarkable. Upper gastroesophagoscopy revealed a smooth, benign appearing stricture at 36 cm beyond which the mucosa was normal. There was a 1.5 cm diameter smooth mucosal bulge noted in the fundus of the stomach just beyond the gastroesophageal (GE) junction. Biopsy form of the bulge was taken and was reported as mild chronic gastritis with Helicobacter pylori infestation. He was treated with proton pump inhibitors for six weeks. 

**Figure 1:**
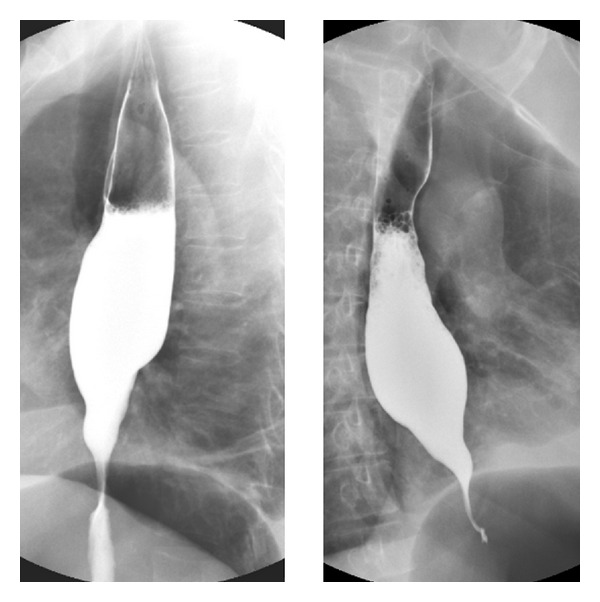


**Figure 2:**
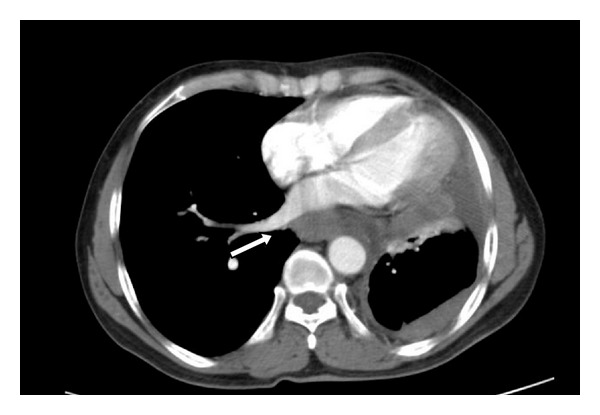


**Figure 3:**
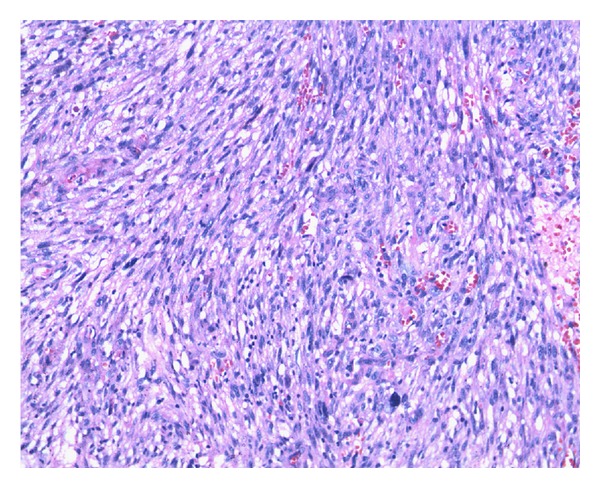


The dysphagia worsened within a span of ten months, and repeat endoscopy revealed a stricture at the same place. Biopsy was not suggestive of malignancy. Barium swallow revealed dilated thoracic esophagus with smooth tapered narrowing (Figure 1). CT scan was done which revealed a lower esophageal wall thickening of 11 mm from T9 to GE junction (Figure 2) with periesophageal soft tissue mass and periesophageal, lesser omental, and peripancreatic nodes along with left pleural effusion and adjacent lung atelectasis. Pleural fluid cytology and pleural biopsy did not show any evidence of malignancy. He was planned for stricture resection and exploratory laparotomy. Peroperatively, lower esophageal thickening was noted and a large, friable, periesophageal tumour mass extending along the crus of the diaphragm involving the coeliac nodes was found. There were multiple enlarged friable fleshy nodes. Multiple biopsies were taken from the perioesophageal tissue and nodal mass, and feeding jejunostomy was done. Histopathologically, fibroadipose tissue with a cellular tumour composed of fascicles of spindle shaped cells with plump oval to elongated, moderately pleomorphic, mitotically active nuclei (4-5 per 10 high power fields) was found (Figure 3). Many haemosiderophages, foci of recent haemorrhage, hyalinization, and congestion were present; the tumor was focally positive for Vimentin and was negative for SMA, CD 34, CAM 5.2, cytokeratin, desmin, S100, and CD 117. Hence, a diagnosis of malignant spindle cell tumor with sarcoma being a possibility was made. 

After literature search, the decision was made to treat as sarcoma esophagus with palliative intent as no formal protocols were available. He received radiation therapy of 46 Gy in 23 fractions by AP-PA portals using Telecobalt machine. CT scan done 6 weeks after radiotherapy showed stable disease. He received 4 cycles of chemotherapy with Doxorubicin (50 mg/m^2^, Day1) and Ifosfamide (5000 mg/m^2^, Day 1) given at 3 weekly intervals. Third cycle was given at a delayed date with reduced dose due to earlier neutropenic sepsis and herpes zoster. 

Repeat CT scan after 4 cycles of chemotherapy showed stable disease. In view of hematological toxicities he had, further chemotherapy was not given. The patient was explained regarding further options—continuing chemotherapy with increased risk of side effects and lesser response rate versus best supportive care, and he opted for best supportive care. Endoscopic stenting was done as palliation. He expired after six months at hometown due to stent blockage or gastrointestinal obstruction beyond the stent. He has had a survival of 20 months from the time of diagnosis with a good quality of life without dysphagia.

## 3. Discussion

### 3.1. Epidemiology and Incidence

Esophageal sarcomas are a rare entity [7]. Morphological variants of esophageal sarcomas as reported in the literature are summarized in Table 1. 

### 3.2. Clinical Features

The median age at diagnosis for esophageal sarcoma is 58 years [26–76 years] [17]. Usually patients present with progressive dysphagia [18], weight loss [18], chest discomfort [19], burning retrosternal pain [20], nausea, and vomiting [20].

### 3.3. Diagnosis

Endoscopically, these are characterized by polypoid and exophytic masses [21] and rarely as ulcerating tumour [22]. Barium studies may show large intramural mass with ulceration/tracking, expansile intraluminal masses, or areas of luminal narrowing [23]. Stricture esophagus is a rare presentation. CT/MRI imaging may show inhomogenously enhancing intramural mass [23]. One of the indications for endoscopic ultrasound and its guided biopsy or fine needle aspiration cytology is submucosal esophageal tumors which otherwise may need open biopsy for diagnosis. This in turn may reduce the time of delay in diagnosis [24].

### 3.4. Pathology

Considering histological appearance and immunohistochemistry of sarcomas of esophagus, carcinosarcomas has both carcinomatous and sarcomatous component. The sarcomatous component of carcinosarcoma is composed of dense interlacing bundles of spindle-shaped cells in the submucosa [25]. 

Pseudosarcoma shows squamous cell carcinoma and mesenchymal components without a transitional zone [19]. 

Synovial sarcoma has undifferentiated spindle cells similar in appearance to synovial sarcoma in other areas but overt mesenchymal differentiation showing smooth muscle, cartilage, or bone formation. X; 18 translocation is a sensitive marker and is demonstrated in 70 to 90% of synovial sarcomas. The specific t(X; 18) (p11.2; q11.2) results in the fusion gene product SYT-SSX [14]. 

Leiomyosarcoma has elongated cells forming interlaced bundles. Leiomyosarcoma constitutes about 0.5% of the malignant neoplasms of the oesophagus [12]. Oesophageal leiomyosarcoma possibly arises from the muscularis mucosa [3]. 

Liposarcoma has the lipogenic component containing giant multinucleated lipoblasts with multiple cytoplasmic lipid vacuoles giving grape-like appearance. The nonlipogenic component can resemble pleomorphic and/or myxoid MFH expansion of Malignant Fibrous Histiocytoma, a round cell sarcoma, a spindle liposarcoma, and/or an epithelioid carcinoma [13]. 

Histiocytic sarcoma has medium-sized tumor cells, indistinguishable from reactive histiocytes with moderate pleomorphism. 

The tumor in our case was unique in that it did not have carcinomatous component and it was focally positive for Vimentin not making it any of the above-mentioned sarcomas of esophagus.

### 3.5. Treatment

Surgery, wherever possible, remains to be the mainstay of treatment [23]. Oesophagectomy/oesophagogastrectomy is the surgery of choice. Even if metastases are present, a palliative resection can still be performed [26]. Endoscopic resection is another surgical option available [27]. The role of adjuvant radiotherapy and chemotherapy is controversial [23]. Palliative procedures like stenting to relieve dysphagia improve quality of life [28].

### 3.6. Prognostic Factors

Factors affecting survival included completeness of resection, growth pattern, postsurgical stage, tumour grade, and tumour location [18]. A rare case of spontaneous regression of oesophageal leiomyosarcoma is reported [29]. The more favourable prognosis associated with carcinosarcoma versus other oesophageal neoplasms has been attributed to early onset of symptoms, resulting in prompt diagnosis and a lower propensity for tumour invasion [30]. As in typical squamous cell carcinoma, early detection and treatment by surgical resection are needed to produce significant long-term survival [31].

### 3.7. The Present Case in the Context of the Literature

Sarcoma is a rare entity among all esophageal malignancies. It presents as an exophytic mass, and in this case, it has presented as a stricture esophagus. Most of these tumors present in locally advanced and disseminated condition, one of the reasons being difficulty and hence delay in diagnosis. Inspite of best efforts, a group among them remains to be histologically uncharacterized. Here, we report a case of malignant spindle cell tumor of esophagus, a cause for a stricture esophagus. A definitive histopathological diagnosis could not be achieved. 

Regarding treatment, there is a role of palliative resection even in case of inoperable disease. In view of locoregional failure, the role of aggressive local treatment should be emphasized.

## Figures and Tables

**Table 1:** Morphological variants of esophageal sarcoma.

Type	Immunohistochemistry	Incidence (among esophageal cancer)	Survival	Reference
1. Carcinosarcoma	Positive for cytokeratin, vimentin, smooth muscle actin, and p53 [10]	Approximately 6%	DFS* of 45 months	Nakagawa et al., [11]
2. Leiomyosarcoma	Strongly positive for SMA, negative for cytokeratin [12]	0.5%	DFS* of 14 months, survival of 20 months	Adad et al., [12]
3. Liposarcoma	Positive only for S100	Very rare; nearly 13 to 15 reported cases	Not mentioned	Garcia et al., [13]
4. Synovial sarcoma	Biphasic morphologic findings positive for vimentin, epithelial (EMA, CK7, AE1/3), bcl-2, and neuroectodermal (CD56, CD57, CD99)X;18 translocation on FISH [14]	Very rare; nearly 10 cases reported	Not known	Butori et al., [15]
5. Myxofibrosarcoma	Positive for CD34, smooth muscle actin, negative for S-100, C-kit, and desmin [4]	Very rare; 1 to 2 cases reported	Not known	Song and Miller, [4]
6. Ewing's sarcoma	MIC2/CD99 positive	Very rare; 1 to 2 cases reported	Not known	Maesawa et al., [5]
7.Granulocytic sarcoma	Subepithelial dense deposits of myeloid cells histologically	Very rare	Not mentioned	Ibrarullah et al., [6]
8.Histiocytic sarcoma	Positive for CD68 and negative for CD1a and CD35, negative for Ki-1 antigen and T-cell and B-cell lineage markers [7]	Very rare	1 month	Akishima et al., [7]
9. Schwannoma	Positive for S100 and vimentin; negative for CD117	Very rare	Not known	Sanchez et al., [8]
10. Rhabdomyosarcoma	Intracytoplasmic cross striations histologically	Very rare;15 reported cases	Not known	Batoroev and Nguyen, [9]
11. Epithelioid sarcoma	Positive for both epithelial and mesenchymal markers, such as cytokeratin, epithelial membrane antigen (EMA), vimentin and CD34	Very rare	Not mentioned	Maggiani et al., [16]

*disease-free survival.

## References

[1] Golioto M, McGrath K (2001). Primary lymphoma of the esophagus in a chronically immunosuppressed patient with hepatitis C infection: case report and review of the literature. *American Journal of the Medical Sciences*.

[2] Ruppert-Kohlmayr AJ, Raith J, Friedrich G, Regauer S, Preidler KW, Szolar DH (1999). Giant liposarcoma of the esophagus: radiological findings. *Journal of Thoracic Imaging*.

[3] Bonavina L, Fociani P, Asnaghi D, Ferrero S (1998). Synovial sarcoma of the esophagus simulating achalasia. *Diseases of the Esophagus*.

[4] Song HK, Miller JI (2002). Primary myxofibrosarcoma of the esophagus. *Journal of Thoracic and Cardiovascular Surgery*.

[5] Maesawa C, Iijima S, Sato N (2002). Esophageal extraskeletal Ewing’s sarcoma. *Human Pathology*.

[6] Ibrarullah M, Sambasivaiah K, Kumaraswamy Reddy M, Wagholikar G (2003). Granulocytic sarcoma of esophagus. *Gastrointestinal Endoscopy*.

[7] Akishima Y, Akasaka Y, Yih-Chang G (2004). Histiocytic sarcoma with fatal duodenal ulcers. *Pathology Research and Practice*.

[8] Sánchez A, Mariángel P, Carrasco C, Venturelli A, Vera G (2004). Malignant nerve sheath tumor of the esophagus (malignant esophageal schwannoma). *Gastroenterologia y Hepatologia*.

[9] Batoroev YK, Nguyen GK (2006). Esophageal rhabdomyosarcoma: report of a case diagnosed by imprint cytology. *Acta Cytologica*.

[10] Kashiwabara K, Sano T, Oyama T (2001). A case of esophageal sarcomatoid carcinoma with molecular evidence of a monoclonal origin. *Pathology Research and Practice*.

[11] Nakagawa S, Yabusaki H, Tanaka O (2009). Rapid-growth carcinosarcoma of the esophagus arising from 0-IIc squamous cell carcinoma after definitive chemoradiotherapy: a case report. *Esophagus*.

[12] Adad SJ, Etchebehere RM, Hayashi EM (1999). Leiomyosarcoma of the esophagus in a patient with chagasic megaesophagus: case report and literature review. *American Journal of Tropical Medicine and Hygiene*.

[13] Garcia M, Buitrago E, Bejarano PA, Casillas J (2004). Large esophageal liposarcoma: a case report and review of the literature. *Archives of Pathology and Laboratory Medicine*.

[14] Billings SD, Meisner LF, Cummings OW, Tejada E (2000). Synovial sarcoma of the upper digestive tract: a report of two cases with demonstration of the X;18 translocation by fluorescence in situ hybridization. *Modern Pathology*.

[15] Butori C, Hofman V, Attias R, Mouroux J, Pedeutour F, Hofman P (2006). Diagnosis of primary esophageal synovial sarcoma by demonstration of t(X;18) translocation: a case report. *Virchows Archiv*.

[16] Maggiani F, Debiec-Rychter M, Ectors N, Lerut A, Sciot R (2007). Primary epithelioid sarcoma of the oesophagus. *Virchows Archiv*.

[17] Rocco G, Trastek VF, Deschamps C, Allen MS, Miller DL, Pairolero PC (1998). Leiomyosarcoma of the esophagus: results of surgical treatment. *Annals of Thoracic Surgery*.

[18] Kwatra KS, Prabhakar BR, Jain S, Grewal JS (2003). Sarcomatoid carcinoma (carcinosarcoma) of the esophagus with extensive areas of osseous differentiation: a case report. *Indian Journal of Pathology and Microbiology*.

[19] Kubota S, Morita T, Murakawa K (1999). A case or pseudosarcoma associated with type 3 squamous cell carcinoma of the esophagus: report of a case. *Surgery Today*.

[20] Liakakos TD, Troupis TG, Tzathas C (2006). Primary liposarcoma of esophagus: a case report. *World Journal of Gastroenterology*.

[21] Kayaselçuk F, Tuncer I, Toyganözü Y, Bal N, Özgür G (2002). Carcinosarcoma of the stomach. *Pathology and Oncology Research*.

[22] Chino O, Kijima H, Shimada H (2000). Clinicopathological studies of esophageal carcinosarcoma: analyses of its morphological characteristics using endoscopic, histological, and immunohistochemical procedures. *Endoscopy*.

[23] Pramesh CS, Pantvaidya GH, Moonim MT, Jambhekar NA, Sharma S, Deshpande RK (2003). Leiomyosarcoma of the esophagus. *Diseases of the Esophagus*.

[24] Ball ABS, Fisher C, Pittam M, Watkins RM, Westbury G (1990). Diagnosis of soft tissue tumours by Tru-Cut biopsy. *British Journal of Surgery*.

[25] Kinoshita Y, Tsurumaru M, Udagawa H (1998). Carcinosarcoma of the esophagus with metastases showing osteosarcoma: a case report and review of the literature. *Diseases of the Esophagus*.

[26] Kimura H, Konishi K, Kawamura T (1999). Esophageal sarcomas: report of three cases. *Digestive Surgery*.

[27] Suwa T, Hori M, Yoshida M (2008). Esophageal leiomyosarcoma: a case treated by endoscopic resection. *Esophagus*.

[28] Conio M, Blanchi S, Filiberti R, De Ceglie A (2010). Self-expanding plastic stent to palliate symptomatic tissue in/overgrowth after self-expanding metal stent placement for esophageal cancer. *Diseases of the Esophagus*.

[29] Takemura M, Osugi H, Tokuhara T, Kinoshita H, Higashino M (1999). Case of spontaneous regression of metastatic lesions of leiomyosarcoma of the esophagus. *Diseases of the Esophagus*.

[30] Ziauddin MF, Rodriguez HE, Quiros ED, Connolly MM, Podbielski FJ (2001). Carcinosarcoma of the esophagus—pattern of recurrence. *Digestive Surgery*.

[31] Madan AK, Long AE, Weldon CB, Jaffe BM (2001). Esophageal Carcinosarcoma. *Journal of Gastrointestinal Surgery*.

